# Supplementary material for: State-specific morphological deformations of the lipid bilayer explain mechanosensitive gating of MscS ion channels
Source: eLife. 2023 Jan 30;12:e81445. doi: 10.7554/eLife.81445 (PMC9925053; doi:10.7554/eLife.81445)
Supplement: Supplementary file 1. — EM maps and atomic models have been deposited in the Electron Microscopy Data Bank (accession number EMD-27337) and the Protein Data Back (entry code 8DDJ). [file elife-81445-supp1.docx]

| Wild-type MscS Open | |
| --- | --- |
| EMDB accession number | EMD-27337 |
| Number of movies | 1,404 |
| Magnification | 130,000 |
| Total electron dosage e^-^/Å^2^ | 50 |
| Pixel size (Å) | 0.532 |
| Voltage (kV) | 300 |
| Defocus range (µm) | 1-2.5 |
| Initial particles | 849,995 |
| Final particles | 43,929 |
| Symmetry imposed | C7 |
| Map resolution (Å) (0.143) | 3.1 |
| *Model refinement* | |
| PDB ID | 8DDJ |
| Non-hydrogen atoms | 14903 |
| Protein residues | 1960 |
| Ligands | 0 |
| *B-factors* (Å^2^) | |
| Protein | 88.73 |
| *Validation* | |
| MolProbity score | 1.99 |
| Clashscore | 6.14 |
| Poor rotamers (%) | 2.14 |
| *Ramachandran plot* | |
| Favored (%) | 93.94 |
| Allowed (%) | 4.62 |
| Outliers (%) | 1.44 |
